# Building houses and managing lawns could limit yard soil carbon for centuries

**DOI:** 10.1186/s13021-019-0124-x

**Published:** 2019-08-16

**Authors:** Morgan E. Peach, Laura A. Ogden, Eleni A. Mora, Andrew J. Friedland

**Affiliations:** 1 Ecology, Evolution, Ecosystems & Society Program, Dartmouth College, Hanover, NH 03755 USA; 2 Environmental Studies Program, Dartmouth College, Hanover, NH 03755 USA; 3 Anthropology Department, Dartmouth College, Hanover, NH 03755 USA

**Keywords:** Lawn, Carbon, Nitrogen, Land use, Management, Legacies, Soil

## Abstract

**Background:**

Comparisons of soil carbon (C) pools across land uses can be confounded by site-specific history. To better quantify the response of soil C pools to residential development and use, we compared yard soils (n = 20) to adjacent mown fields and second-growth forests within land-use clusters (LUC; n = 12). Land uses within clusters shared site-specific legacies (land use and other soil forming history) prior to residential development (15–227 years ago). We analyzed soil cores to 60-cm depth for carbon, nitrogen, and bulk density. Within one LUC, we monitored soil dissolved organic carbon, moisture, and thermal regimes to explain soil C dynamics.

**Results:**

We accounted for pre-development legacies to test how present uses affect soil properties. We found that yard soil C pools to 60-cm depth (9.07 ± 0.32 kg C m^−2^; mean ± SE) were smaller than fields (10.26 ± 0.44 kg C m^−2^) and forests (10.62 ± 0.87 kg C m^−2^). Fields contained more nitrogen to 60-cm depth (0.78 ± 0.043 kg N m^−2^) than yards (0.68 ± 0.030 kg N m^−2^) and forests (0.69 ± 0.057 kg N m^−2^). Time since development predicted decreased yard and field soil C/N, field soil N accumulation, and reduced yard bulk density. In old yards (> 150 years), where residents in recent times mowed monthly to bimonthly and left clippings on the lawn, there was evidence of soil C and N gains relative to old commercially managed yards mown weekly with clippings exported.

**Conclusions:**

Our study suggests land conversion to yard can limit soil C pools for centuries, with contemporary management key to that trajectory. Our research points to the importance of accounting for pre-development legacies to reveal the response of soil properties to land conversion and present use. This work can inform policies and land use intended to enhance the soil C sink and minimize development-related soil C losses.

**Electronic supplementary material:**

The online version of this article (10.1186/s13021-019-0124-x) contains supplementary material, which is available to authorized users.

## Background

Soils contain the largest terrestrial pool of C (1325 Pg to 1 m depth) [1]. Land conversion and use alter soils, creating a mosaic of legacies across urban–rural gradients [2–5]. Soil C pools respond to past and present use [6–10] but the direction and magnitude of this response in rural residential ecosystems is not well known. Previous work focuses on urban and suburban areas [11–17] where heterogeneous legacies, of past use and other site-specific history, can obscure effects of present use [18–20]. In this study, the relative homogeneity of northeastern United States landscape history [21–23] allowed us to account for pre-development legacies and discern the response of soil C pools to land conversion and contemporary use. We studied a yard-field-forest use-intensity gradient, typical of the rural residential northeastern United States temperate forest, to test the degree to which land-use change and present use affect soil C dynamics.

From 1950 to 2000, developed rural lands expanded to fifteen times the area of urban lands in the United States, a 20% increase in rural land cover relative to a 1% increase in urban cover [24]. In the northeastern United States land conversion to residential use occurred at a rate of 6000 ha year^−1^ in 2011, while reforestation was negligible from 1985 to 2011 [25]. Conversion to rural residential land use replaces native or agricultural covers with houses, lawns, and other plantings [26]. Lawns, a managed turfgrass system, cover three times more area in the continental United States than any irrigated crop [27]. Land conversion disturbs soil and releases C to the atmosphere [28]. Initial losses of soil C, dependent upon conversion-related disturbance intensity, can be recovered as soil C accumulates following vegetation establishment [29]. Post land-use conversion the yard soil C pool is dynamic, with C accumulation or loss dependent upon the relative rates of inputs (e.g., primary production, fertilization) and outputs (e.g., respiration, clipping export, leaching) [30, 31]. The magnitude of net annual soil C fluxes, such as pulses of soil respiration counterbalanced by C flow to roots and soil [32, 33], are small compared to the overall soil C pool. Carbon allocation belowground can stimulate decomposition, dissolved organic carbon production, and C release [34], while a fraction of this C can be stabilized via microbial processing [35, 36]. Over centuries, these processes can yield discernable changes in soil C pools.

Residential parcel owners alter vegetation and soils across use-intensity gradients, with a variety of outcomes across soil depths. In intensively used yards, soils are modified by residential development-related excavation that involves heavy equipment. Excavation can mix, import, or export soil, redistributing nutrients across depths [11]. In fields, a less intensively used grassland than lawns, there is limited history of excavation, but mowing could affect bulk density and rooting [37]. Lawn turfgrass roots are concentrated at 0–15 cm [38], while field vegetation is deeper rooted [39]. This could distribute nutrients to greater depths in fields, possibly stabilize C [40], or prime soil C losses [41]. In least intensively used forests, high C/N plant inputs may lead to soil C retention, although labile grassland inputs could stimulate efficient microbial bioaccumulation, turnover, and C stabilization [42]. Across use-intensity gradients, C and N likely diminish with depth, but past and present uses may affect the vertical distribution and pools of nutrients.

Land managers inherit a legacy of past uses and land changes, yet could control soil C dynamics through management [15, 43–45]. Land-use change alters soil properties, microclimate, and manager-plant-animal-microbe assemblages, which affects whether soils are a C source or sink [46, 47]. For example, parcels with woody plants, or yard management involving fertilization or irrigation, can increase soil C pools [13, 48, 49]. Mowing can stimulate lawn soil respiration [33], which exceeded forest soil respiration in the Boston, MA, USA region, and was possibly related to a warm yard microclimate [7]. Soil respiration depletes yard soil C pools over time, if not matched by C inputs via plant productivity or amendment (e.g., compost, lime). Management could alter soil C dynamics, but soil C pools vary across the landscape mosaic as an outcome of contemporary use interacting with soil-forming site history [11, 50].

Climate, a soil-forming factor [50], could explain soil C pool responses to residential development and yard use [51]. However, across cool temperate steppe of Denver, CO, USA [12], warm, temperate Baltimore, MD, USA [13, 49], and cool, temperate Madison, WI, USA [45], researchers found that residential yard soils were more carbon-rich than native soils. Yard use could enlarge soil C pools across varied climates and soils in urban and suburban areas, but there were no site-specific pre-development soils to serve as a reference in former studies. Differences could be due to soil heterogeneity, the product of anthropogenic and biogeophysical pre-development legacies, in addition to present use.

**Fig. 1:**
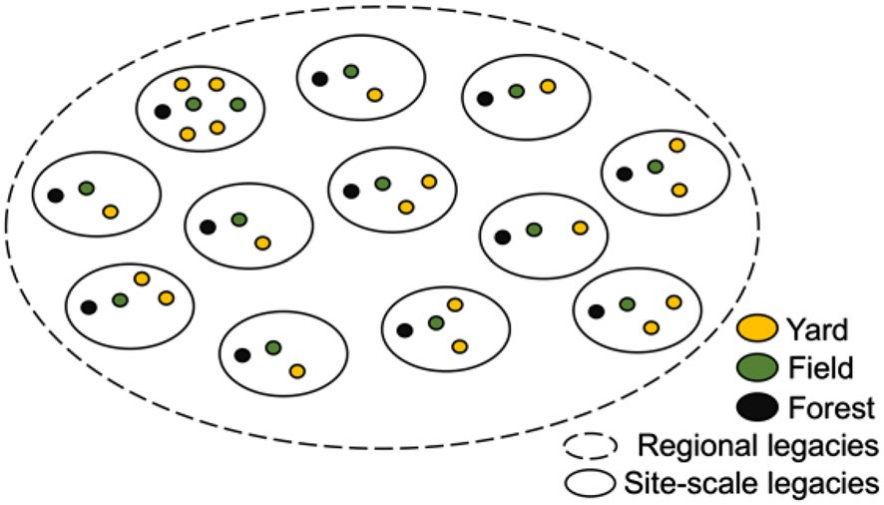
Conceptual diagram of the experimental design to discern the response of slow-changing, heterogenous soil properties to present uses nested within shared pre-development site- and regional-scale legacies

Landscape heterogeneity can yield misleading conclusions about how land conversion and use affect soil C pools. Yards can appear to be a significant C sink compared to native lands when yard site-specific pre-development soil properties are unknown [13, 45, 49, 52–54]. If land conversion to yard occurs on higher quality soils, such as floodplains, soil C losses with development could result in yard soil C pools that exceed native lands elsewhere, where soils could be lower quality and undesirable for development. We therefore used the land-use cluster comparative framework, where soils shared biogeophysical and anthropogenic legacies at the time of residential development (Fig. 1). This accounted for the magnitude of soil C pools prior to present use. If not accounted for, this can confound comparisons of soil properties across present uses. Legacies can overwhelm the effect of present use on slow-changing variables [55]. Our experimental design distinguished the effects of site-specific pre-development legacies and present use on slow-changing, heterogeneous soil properties.

Yards in urban and suburban areas do not share site-scale history with reference lands elsewhere. This can confound understanding of how residential land conversion and use affects soil properties. We therefore devised an experimental design of land-use intensity gradients within clusters that shared similar history. This permitted us to discern the effect of residential development and present use on soil C and N pools. We hypothesized that (1) intensifying use and conversion-related disturbance, within yards and across present uses, would drive soil C losses, and (2) differences in soil properties across use-intensity gradients would be pronounced at the surface and amplify when integrated over depths. We asked the following research questions: How do soil C and N pools differ across yard-field-forest use-intensity gradients? Is varying management intensity, within yards and across uses, associated with changes in soil C and N pools over time since residential development?

## Materials and methods

### Regional legacies prior to residential development

Soils in the New England region of the northeastern United States have been subject to human influence since the last ice age, approximately 13,000 years before present [21, 56]. By the mid-nineteenth century, European settlers cleared 45–65% of the New England forest for agricultural and industrial purposes [57]. Pasture lands covered 70–80% of the landscape for over 100 years, followed by forest regrowth on abandoned lands [58, 59]. By the mid-twentieth century, residential development began to cover old fields and clear second-growth forest [24]. Soils of our study region shared this relatively homogeneous regional land-use legacy, verified through review of archival documents and historic aerial imagery (Additional file 1: Figure S1). Regional-scale pre-development legacies altered soils similarly, but site-specific differences in history could confound comparisons of slow-changing soil properties across present uses.

### Site selection

We sought parcels for participation that represented yard-field-forest use-intensity gradients, of known history, across a broad range of times since residential development. We therefore selected parcels spanning New Hampshire and Vermont, USA, of the Upper Connecticut River Valley [60], a cool temperate forested region (mean annual 28 °C; 840 mm precipitation) [61], where old homes remained as single-family residences, with documented land-use histories (e.g., historical societies, state archives, local libraries) that varied across sites, but shared similar regional soil-forming anthropogenic and biogeophysical legacies.

We selected clusters of parcels to include in our sample with the contiguous land uses of yards, mown field, and second-growth forest (Fig. 1). Present-day land uses within “land-use clusters” (LUC) shared site-specific history (anthropogenic and biogeophysical) prior to present use. LUC allowed us to draw comparisons between present uses on the backdrop of a shared site-specific pre-development legacy, within a similar regional legacy reference frame. We sought participation from homeowners (> 10 years residency) living in a broad range of house ages (15–227 years) to reveal a cumulative effect of longer-term management and time since residential development on slow-changing soil properties. Each participating household resided on a parcel (> 0.53 ha) with similar patterns of lawn (> ~ 90% of yard cover), garden, and woody vegetation adjacent to old fields and second-growth forest, which is common in rural temperate forest residential development [15, 26, 62]. Our sample included 20 residences across twelve LUC.

We sampled twenty yards to discern the effect of varying yard management intensity on soil properties, and sampled fields and second-growth forest within land-use clusters to account for site-specific pre-development legacies and draw comparisons across a use-intensity gradient. Fields served as a less intensively managed grassland comparison with yards. To the extent possible, clusters with annually mown fields (n = 11) of limited anthropogenic nutrient import or export were selected for sampling instead of fields in current use for hay (n = 1) or pasture (n = 1). Fields sampled persisted since European settlement, which we confirmed by cross-referencing 1930’s aerial photography with interviews and archival documents (Additional file 1: Figure S1).

### Homeowner interviews and yard management intensity categories

We conducted semi-structured interviews of homeowners (in person; 20 interviews; 1–1.5 h. each) about their parcel history and management in the spring of 2016. Interviews with homeowners supplemented our land-use legacy archival research at local libraries and town historical societies. We asked homeowners about regional and parcel history across yard, field, and forest, during and before their time of residency. This included questions about planting choices, house age and history, disturbance events (e.g., storms, excavation), above- and belowground infrastructure, yard management, livestock, pets, wildlife, and gardening. These categories of questions documented site to regional-scale legacy effects and present use.

Homeowners lived in a range of house ages (15–227 years; mean: 101 ± 18.8 years), each occupied by participants for at least 10 years (mean: 26 ± 2.6 years). Given the relatively limited sociodemographic diversity of our study region, we expected yard management to be homogeneous compared to findings of heterogeneity across the United States [63]. Homeowner interviews revealed differences in yard management that we hypothesized would affect soil C and N pools. We used these differences as analytic categories, as we discuss below.

Following interviews, we sorted participants into yard management intensity categories. Yard management included mowing, irrigation as needed, and annual to sporadic fertilizing, but differed based on whether homeowners hired a “commercial manager” (n = 9) who mowed weekly and removed clippings, or were “do-it-yourself” (n = 11) who mowed bimonthly to monthly and rarely removed clippings (see 66 for similar yard management intensity categories). Eleven homeowners managed old yards (56–227 years) while nine homeowners managed young yards (15–43 years), with yard ages distributed evenly across management intensity categories. Yard management activities, other than mowing and clipping export, were inconsistent and randomly distributed across our sample.

Decades following house construction and yard establishment, we expected young yard soils to vary in response to residential development in addition to pre-development legacies. We expected the effect of modern residential development to overwhelm the incremental, cumulative effect of yard management in these recently developed soils. Our interviews suggested residents that hired commercial managers were more likely to manipulate their young yard soils than do-it-yourself residents during and following development (e.g., loam import, fertilization) to establish an aesthetically pleasing lawn. We therefore assumed baseline soil properties varied across yards in response to residential development practices and were associated with yard management intensity categories. We assumed old yard soils, that were not subject to modern excavation, had recovered from residential development centuries ago, increasing the likelihood of discerning yard management effects on soil properties. We therefore expected old yard soils to vary in response to different yard management intensities over the past decades.

### Soil analyses within land-use clusters

We sampled 12 land-use clusters (LUC) in the summers of 2016 and 2017, across 20 yards, 13 fields, and 12 forests. Prior to sampling, soils were assessed via probing and the texture-by-feel method [65] to ensure soils of a present use were not clay rich relative to adjacent uses within a cluster. While we did not expect to observe clay-rich soils in our study region, high clay content in one land use could alter soil C dynamics to an extent that confounds soil C pool comparisons across clustered uses, and would have suggested different within-cluster soil-forming legacies (Additional file 1: Table S1). We did not control for potential soil mixing or import of loam to yards, which can occur in modern excavation associated with residential development and lawn establishment. Our field-based soil texture characterizations agreed with high resolution soil survey geographic database maps (SSURGO), based on USDA National Cooperative Soil Surveys [66]. In yards, we sampled soils within the predominant lawn cover. We extracted three soil cores to 60-cm depth at random locations within a randomly placed 16 m dia. plot to represent each land use within a cluster, while avoiding belowground infrastructure. We extracted soil cores (dia. 7.3 cm) from 0 to 10 cm and 10 to 20 cm depth, and augur extracted (dia. 4.5 cm) soil from the 20 to 40 cm and 40 to 60 cm depths. We estimated the volume of augured deep soil by volumetric backfilling with medium-grain sand. We collected 135 soil cores to 60-cm depth.

We air-dried soils to stable mass under ambient laboratory conditions, and then processed samples following standard protocols [67]. We passed air-dried soils through a 2-mm sieve, separating roots and rocks from the < 2 mm fraction to obtain a homogenous representative sample. We washed roots from the upper 20-cm of mineral soil on a 250-μm sieve for 1 min with deionized water, air-dried, and massed them. We massed rocks, and estimated the volume of rocks within each sample using an average particle density of 2.65 g cm^−3^. To estimate soil bulk density, we oven-dried a 15.0 g air-dried subsample at 105 °C for 48 h to calculate the oven-dry mass and moisture content of bulk soil. We subtracted rock mass and volume from the oven-dry mass and volume of soil within each depth increment to calculate bulk density. We collected a separate 3.0 g soil subsample, picked it free of roots, ground, and oven-dried it at 60 °C for 24 h prior to measurement of soil CN concentrations with a Costech ECS 4010 Elemental Analyzer (Costech Analytical Technologies Inc., Valencia, CA). We measured the pH of a 6.0 g soil subsample, composited by land use and depth increment, in a slurry of 12 mL of deionized water. To remove inorganic carbon, we acidified soil samples of pH 6 or greater (across depths: 59 samples from 11 yards, 13 samples from 2 forests, 3 samples from 2 fields) with 6 M HCl, and analyzed the sample for remaining organic carbon. We calculated the inorganic carbon fraction by subtracting organic carbon from total soil carbon concentrations, and total soil carbon and nitrogen pools according to the following equation:

$$S \, = \, CN \times Bd \times V \times Hf$$

where, *S*: soil carbon or nitrogen pool (kg C or N m^−2^), *CN*: C or N concentration (kg C or N kg soil^−1^), *Bd*: Bulk density (kg m^−3^), *V*: Volume of sampled soil of 1 m^2^ surface area, and specific depth, *Hf*: (1 − (stone volume + root volume)/V) [67]. We estimated stone and root volumes (m^3^) via previously described methods. Given the small root masses collected (mean 3.8 g over 0–20 cm depth), we did not include root volume in the soil C or N pool calculation. We estimated soil C and N pools using the mean of three un-composited cores per depth increment within a land-use site, and summed across depth increments to 20, 40, and 60-cm depth. We encountered an impenetrable layer at 40-cm depth in four yards that were omitted from analyses to 60-cm depth.

### Microclimate and soil solution along a yard-field-forest gradient

To understand the relationship of soil C pools, microclimate, and soil solution dissolved organic carbon, we intensively studied one land-use cluster in Hanover, NH. This cluster had well documented land-use history, was accessible for intensive study, and shared the soil-forming pre-development legacies, residential development history, and present uses of our study region. We installed Campbell Scientific CR800 dataloggers (Campbell Scientific Inc., Logan, UT) in May 2016, with three CS655 sensors buried vertically in the upper mineral soil (10–15 cm) at random locations within each 16 m diameter plot, continuously logging volumetric water content and temperature. During the 2016 growing season, we sampled soil solution following precipitation events greater than 13 mm (~ every 2 weeks) in the upper (n = 5; 15-cm depth) and deep mineral soil (n = 5; 50-cm depth). We extracted samples from tension lysimeters by applying a vacuum of 70 cbar (Soil Moisture Equipment Corp., Santa Barbara, CA). We filtered samples to 1.1 μm in extraction, which represented a broader spectrum of organic compounds relative to 0.45 μm or 0.7 μm filters often used [68]. We transported soil solution samples to the lab and treated them with 9 M H_2_SO_4_ to pH 2, which halted microbial respiration and removed carbonates. Soil solution samples were stored at 4 °C for up to 3 weeks prior to analysis with a Shimadzu Total Organic Carbon Analyzer (Shimadzu Scientific Instruments, Columbia, MD). We managed the yard according to do-it-yourself management regimes of our study region, which involved mowing every 2 weeks and leaving clippings on the lawn. At this intensive study site, we sampled soil from yard, field, and forest using methods employed at other LUC.

### Statistical analyses

To account for pre-development legacies and test how present uses affect soil properties, we used linear mixed-effects models of R’s *lme4* package [69]. Our experimental design was “naturally nested and partially crossed” [70] with unequal land-use sample sizes overall and within clusters (LUC) that represented site-specific pre-development legacies. We defined LUC as a random effect in linear mixed-effects models to address the issue of non-independence of uses within a cluster. This approach partitioned variance between LUC and present uses while preserving degrees of freedom by not estimating random effect means. Soil response variables within land uses were normally distributed by visual inspection, with equal variances by Levene tests. We report *p*-values (α ≤ 0.05) using restricted maximum likelihood and Satterthwaite approximations of degrees of freedom [71].

We expected soil responses to present use to diminish with depth and as use intensity decreased. We tested whether present use and depth explained variation in soil properties with type-III sums of squares *F*-tests of *lme4* output (*anova* function, *lmerTest* package) [72]. In significant models, we performed contrasts by land use within depth increments. In contrasts, we report Tukey-adjusted *p*-values from *t*-tests of the null hypothesis that least square means differences equal zero (*emmeans* package) [73]. We contrasted least-square means to adjust for the effect of site-specific pre-development legacies. We report and interpret significant interactions before main effects. We represent model fits by reporting marginal (r^2marg^; fixed effects) and conditional (r^2cond^; fixed + random effects) r^2^ values calculated using the *r.squaredGLMM* function of the *MuMIn* package [74, 75].

We expected yard soil C and N pools to respond to management (> 10 years; 26 ± 2.6 years) but the direction and magnitude of this response, after accounting for pre-development legacies and with time since development, was unknown. Therefore, across yard soil depth increments we tested the interaction of management intensity category and time since development (y ∼ time since development*management + depth + (1| LUC)). If the interaction was significant, we performed a *t* test of differences in management intensity category regression line slopes using *emtrends* of the *emmeans* package [73] and report Tukey-adjusted *p*-values. If the interaction was not significant, we tested fixed effects alone. For fields (n = 13) and forests (n = 12) we fit a simple linear model (y ∼ time since development) given levels of LUC (n = 12) matched the number of land-use sites. We performed analyses in R, v. 3.4.4 [76].

## Results

### Yard-field-forest soil comparisons after accounting for pre-development legacies

**Fig. 2:**
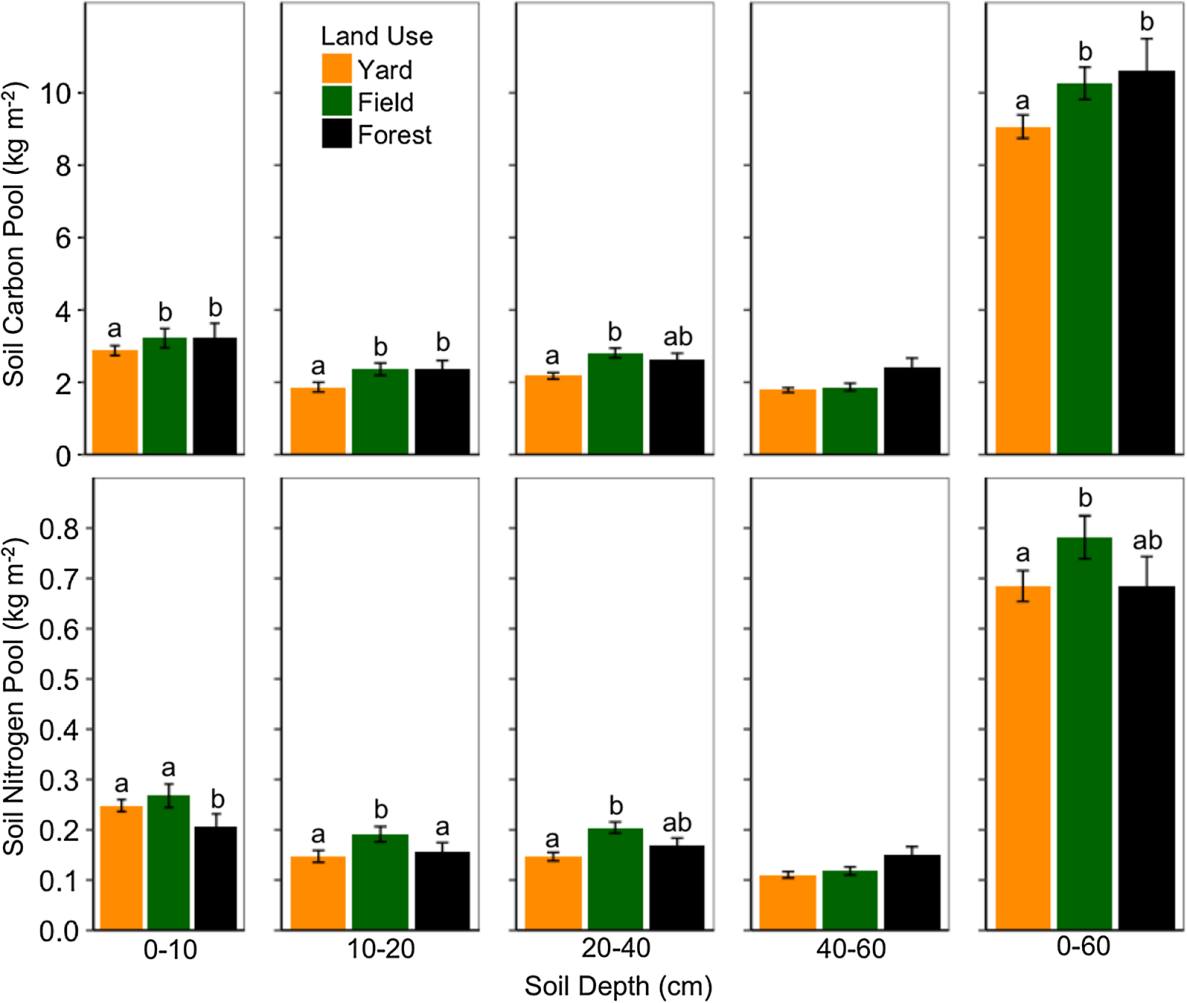
Mean soil carbon and nitrogen pools (± SE) by depth and summed to 60-cm, across yard (n = 20; n = 16, 40–60 and 0–60 cm), field (n = 13), and forest (n = 12) uses comprising a use-intensity gradient within clusters (n = 12). Each land-use site is represented by three soil cores. Letters indicate differences of least-square means that adjust for site-specific pre-development legacies. Yard and field soil C and N pools were significantly different to 40-cm and marginally to 60-cm depth (*p* < 0.10)

We compared yard, field, and forest soil properties across depths. Present use explained variation in soil C pools (r^2marg.^ = 0.27; *p* = 0.0001) and interacted with depth to explain variation in soil N pools (r^2marg.^ = 0.39; *p *= 0.03) (Table 1). After accounting for site-specific pre-development legacies, yards contained 1.8 ± 0.65 kg C m^−2^ less than forests to 60-cm depth (*p* = 0.03), and 1.60 ± 0.41 kg C m^−2^ less than fields to 40-cm depth (*p* = 0.001), but field and forest soil C pools were not different (Fig. 2, Additional file 1: Table S2). To 10-cm depth, yard and field soil N pools were 1.25 times larger than forest (*p* = 0.08, *p* = 0.008, respectively), but not different from each other. To 40-cm depth, fields contained 0.13 ± 0.05 kg N m^−2^ more than yards and forests (Additional file 1: Table S2).

**Table 1:** Present land use and depth effects on soil properties after accounting for pre-development legacies

Response	Factor	*F* value (df^a^)	*p* value	Marginal r^2a^	Conditional r^2a^
Soil C Pool	Land use	9.74 (2, 161.9)	0.0001	0.27	0.38
	Depth	19.5 (3, 158.8)	< 0.0001		
Soil N Pool	Land use	6.0 (2, 156.2)	0.003	0.39	0.46
	Depth	29.5 (3, 152.8)	< 0.0001		
	Land use: depth	2.3 (6, 152.9)	0.03		
Soil C/N	Land use	2.6 (2, 156.7)	0.07	0.18	0.23
	Depth	5.2 (3, 152.1)	0.002		
	Land use: depth	2.3 (6, 152.1)	0.04		
Bulk density	Land use	18.3 (2, 155.0)	< 0.0001	0.38	0.47
	Depth	23.2 (3, 151.7)	< 0.0001		
	Land use: depth	4.1 (6, 151.7)	< 0.001		

^a^Denominator degrees of freedom by Satterthwaite approximation. Marginal r^2^ represent the fixed effect of present land use and conditional r^2^ include the random effect of land-use cluster. Results from equation: response ∼ land use + depth + (1| land-use cluster). Fixed effects interaction included when significant

**Fig. 3:**
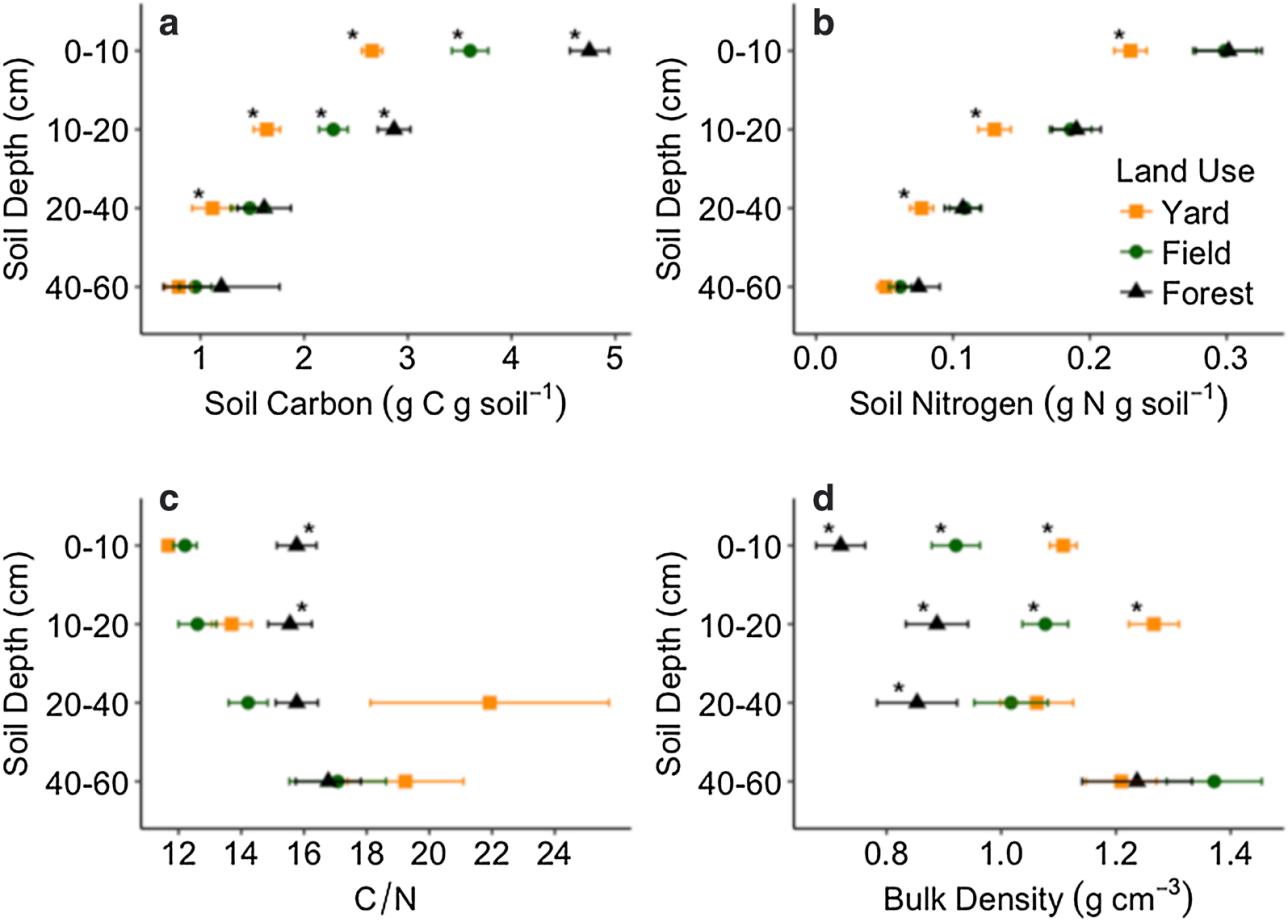
Mean ± SE soil carbon (**a**) and nitrogen (**b**) concentrations, C/N (**c**), and bulk density (**d**) across yards (n = 20; n = 16, 40–60 and 0–60 cm), fields (n = 13), and forests (n = 12). Asterisks indicate differences (*p* < 0.05) of least-square means that adjust for site-specific pre-development legacies. Forest soil bulk density was not different than field at 20–40 cm depth

Present use explained variation in soil C and N concentrations (r^2marg.^ = 0.60; r^2marg.^ = 0.63). Forest soil carbon to 10-cm depth was a factor of 1.8 and 1.3 times more concentrated than yard and field soils (Fig. 3). Yard soil N concentrations were an average 23% lower than fields and forests to 10-cm depth, but field and forest soil N concentrations did not differ. Across uses, soil C and N concentrations were more similar with depth.

Land-use clusters represented a shared pre-development biogeophysical and anthropogenic legacy of soils within a cluster prior to present use. Land-use clusters explained 30% and 26% of the variance in soil C and N pools to 60 cm, while present use explained 13% and 8% (Additional file 1: Table S3). Present use, depth, and land-use cluster explained more variation in soil N pools (r^2cond.^ = 0.46) than soil C pools (r^2cond.^ = 0.38) (Table 1).

Lower soil C/N may indicate elevated microbial processing of soil organic matter. Present use interacted with depth to explain variation in soil C/N (Table 1). Yard (11.7 ± 0.16) and field soil C/N (12.2 ± 0.37) were lower than forest (15.8 ± 0.63) to 10-cm depth (*p* < 0.0001; Fig. 3). Present use explained 60% of the variance in soil C/N to 10-cm depth, 36% to 20-cm depth, and did not explain soil C/N averaged over greater depths (Additional file 1: Table S3). Land-use clusters did not explain variation in upper soil C/N, while differences by present use dissipated with depth.

Present use interacted with depth to explain variation in bulk density (Table 1). Present use explained 55% of the variance in soil bulk density averaged over 20-cm depth (*p* < 0.0001) and 23% of the variance to 60-cm depth (*p* = 0.002) (Additional file 1: Table S3). Across depths, yard (1.14 ± 0.04 g cm^−3^) and field soils (1.097 ± 0.04 g cm^−3^) were more compacted than forest soils (0.92 ± 0.06 g cm^−3^). Compaction was pronounced in yard upper soil, intermediate in field, least in forest, and differences by present use dissipated with depth (Fig. 3). Land-use clusters did not explain bulk density to 20-cm depth, but over 60 cm accounted for 21% of the variance (Additional file 1: Table S3).

Across land uses and depths, soils were acidic (mean pH 5.6). Averaged across depth increments, yard soils were less acidic (5.84 ± 0.08) than forest (5.21 ± 0.11) (*p *= 0.01) but not different than field (5.56 ± 0.05). Field and forest soils did not differ in pH. In eleven yards with soil pH > 6, with history of lime and wood ash fertilization, inorganic C accounted for a mean 25% of yard soil C pools.

**Fig. 4:**
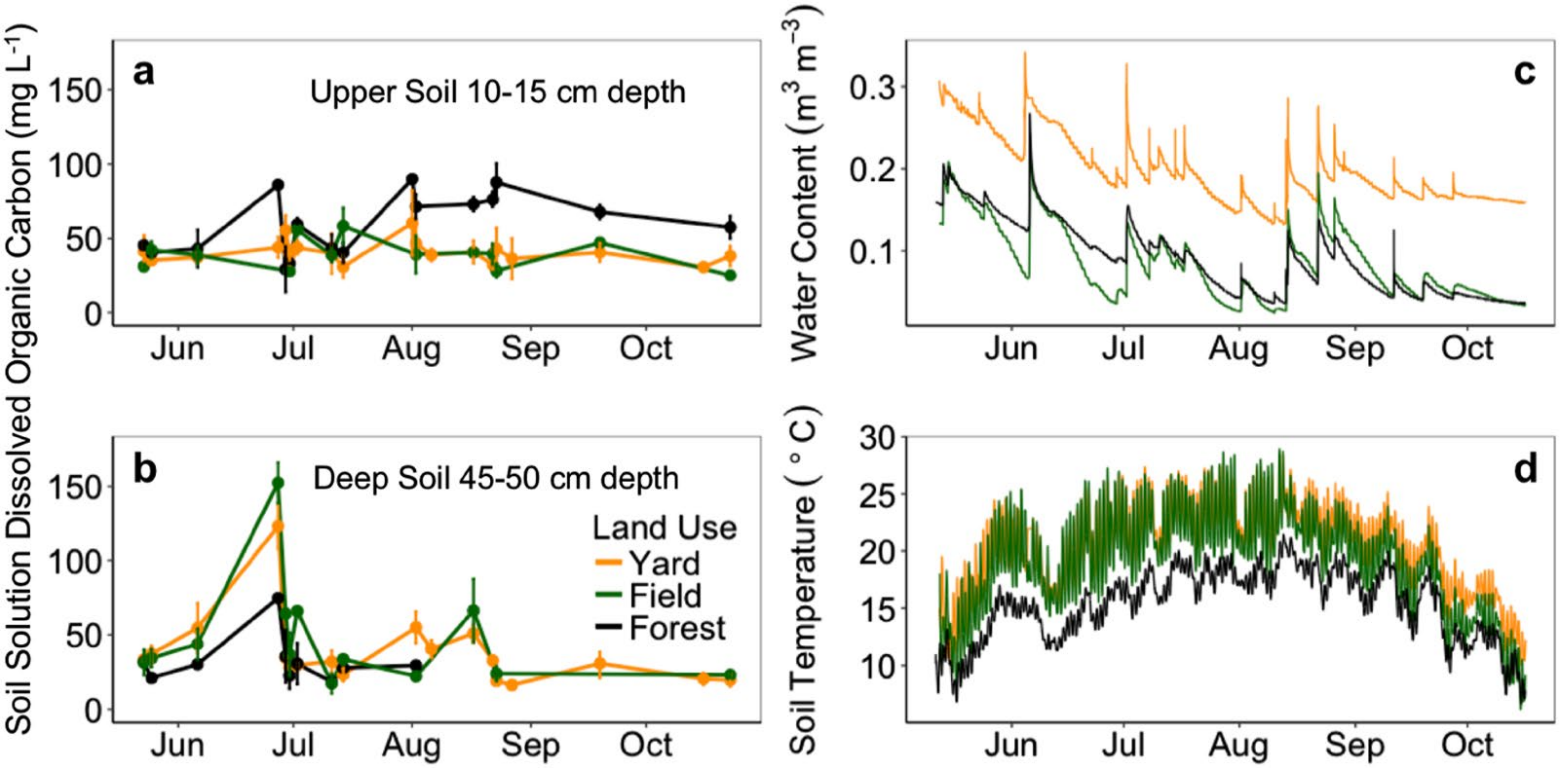
Dissolved organic carbon in upper (**a**) and deep (**b**) soil solution (mean ± SE of three to five samples per date) and upper soil water content (**c**) and soil temperature (**d**) monitored at three locations within each land use that shared site-specific pre-development legacies

Intensive monitoring at one land-use cluster suggested soil solution dissolved organic carbon (DOC) concentrations in yard and field deep soil were an average 5.57 and 13.25 mg L^−1^ greater than forest (Fig. 4). Early in the growing season we observed a pulse of DOC in yard and field deep soil (45–50 cm depth), an average 48.4 and 77.7 mg L^−1^ greater than forest deep soil. Across samples dates, forest upper soil (10–15 cm) DOC concentrations were 40.4 ± 2.14 mg L^−1^ relative to 41.0 ± 1.74 mg L^−1^ in yard and 69.3 ± 8.3 mg L^−1^ in field. In the late growing season, forest deep soil was too dry to extract soil solution. Yard soils were an average 5.1 °C warmer and 0.11 m^3^ m^−3^ wetter than forest soils at this site. As is visually evident (Fig. 4), forests soils were buffered relative to variable soil thermal regimes in yard and field.

### Changes in soil properties with time since residential development

We developed models to discern changes in soil properties across yard-field-forest use-intensity gradients with time since residential development. In yards, which are more intensively used than fields and forests, time since residential development predicted decreased soil C/N and bulk density, but did not explain other soil properties (Additional file 1: Table S4). Changes in yard soils over time since development could have been concealed by differences due to yard management.

We tested the interaction of yard management intensity category and time since residential development. Our sample included two groups of yard managers (do-it-yourself or commercial manager) that managed lawns consistently throughout the residency of homeowners (mean: 26 ± 2.6 years). In old yards (56–227 years) the duration of homeowner residency was a fraction of yard history, but we assumed old yard soils had recovered from residential development and were not impacted by modern excavation, increasing the likelihood of discerning yard management effects on slow-changing soil properties. In young yards (15–43 years), we expected soils were recovering from residential development and modern excavation, and the cumulative effect of lawn management was therefore less likely to be evident.

We assumed that baseline soil properties varied across yards at the time of residential development and could be associated with lawn management intensity categories. We expected homeowners that hired commercial managers were more likely to manipulate yard soil properties immediately following development (e.g., loam import, fertilization) relative to do-it-yourself residents, which our interviews corroborated. We therefore allowed intercepts to vary by lawn management intensity category when testing the interaction with time since residential development (for comparison, see Additional file 1: Figure S2 with a shared intercept representing the alternative assumption that yards shared baseline soil properties following residential development). Nonetheless, given the scalar mismatch between the duration of lawn management we documented and time since development in old yards, the directionality of differences by management category, as opposed to magnitude of estimates we report, should be interpreted.

**Fig. 5:**
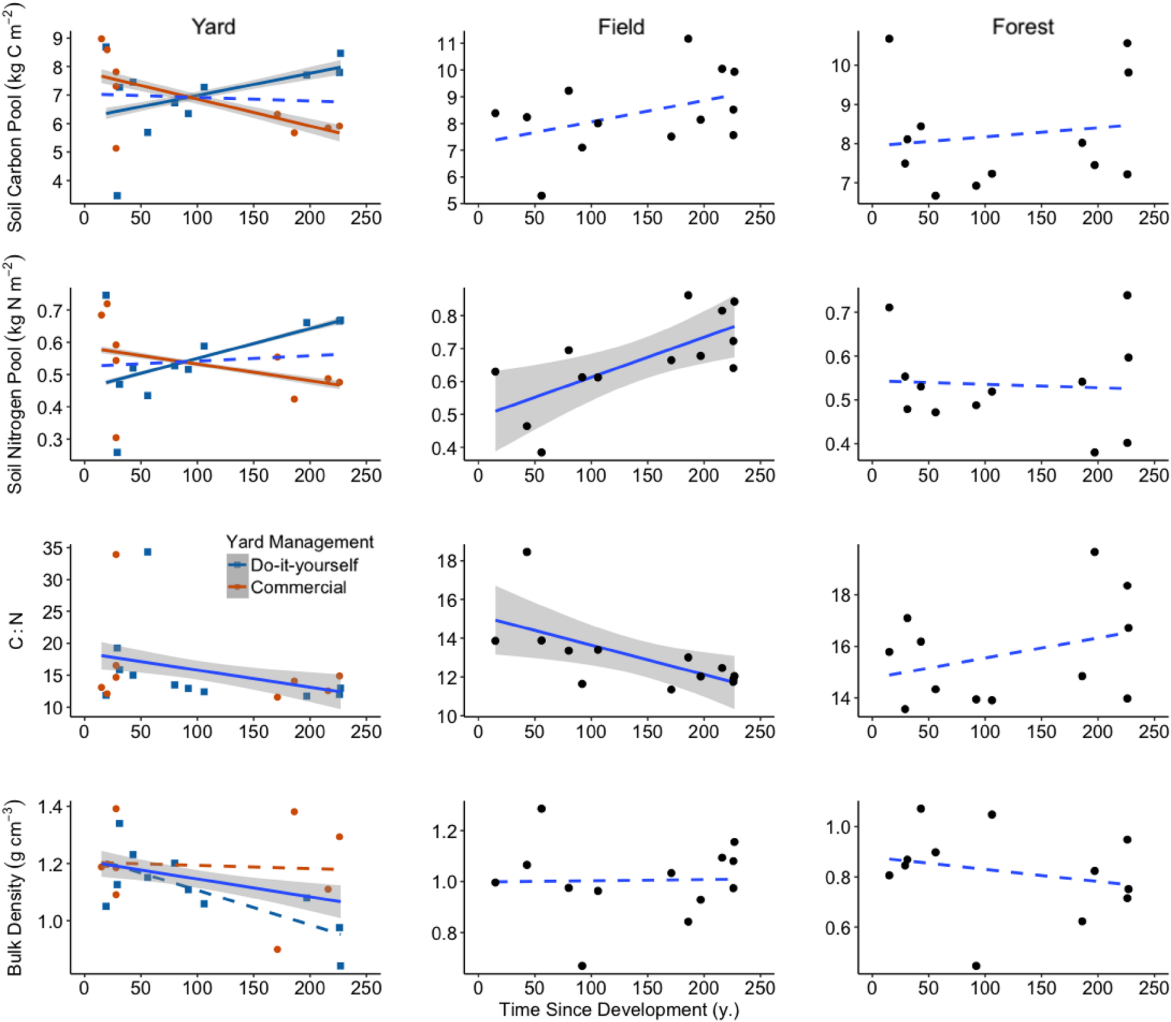
The association of soil C and N pools, C:N, and bulk density with time since development across yard, field, and forest soils to 40-cm depth. Each point represents the mean of three cores per land-use site. Predictions of C:N and bulk density apply to all yards. Shaded areas are confidence intervals of a significant model, dashed lines are non-significant

The interaction of yard management intensity categories with time since development predicted yard soil C and N pools (*p* = 0.04) (Additional file 1: Table S4). The response of soil C and N pools to residential development and yard management were coupled. Do-it-yourself managed yards, that were mown monthly to bimonthly and in which clippings were not removed, were associated with a 0.018 ± 0.007 kg C m^2^ year^−1^ (t_11.3_ = 2.48, *p* = 0.03) and 0.0015 ± 0.0007 kg N m^2^ year^−1^ (t_10.55_ = 2.14, *p* = 0.06) increase to 40-cm depth relative to commercially managed yards (difference of slopes; Fig. 5). Commercially managed yards, that were mown weekly and clippings exported, were associated with losses of 0.009 ± 0.005 kg C m^2^ year^−1^ and 0.0005 ± 0.0004 kg N m^2^ year^−1^ since development to 40-cm depth.

Site-specific pre-development legacies, represented by land-use clusters, explained 27% of the variance in yard soil C pools to 60-cm, but didn’t explain variance in upper soil C or N pools. The interaction of yard management intensity categories with time since development explained 24–29% of the variance in soil N pools from 60 to 20-cm depth, but land-use cluster did not explain additional variance.

The interaction of yard management intensity categories and time since development marginally explained bulk density across depths (*p* = 0.09) (Additional file 1: Table S4). Yard management and time since development interacted to predict bulk density to 10-cm depth (*p *= 0.0006; r^2marg.^ = 0.52). With time since development, commercially managed yards were compacted by 0.0018 ± 0.0004 g cm^−3^ year^−1^ to 10-cm depth relative to do-it-yourself managed yards that were mown monthly to bimonthly (t_16_ = − 4.28; *p* = 0.006; difference in slopes). Yard management and time since development interacted to explain less variance in bulk density with depth, but still had an effect averaged over 60-cm depth (*p *= 0.06; r^2marg.^ = 0.33). Land-use cluster, representative of site-specific pre-development legacies, did not explain variance in yard soil bulk density since development.

Lower soil C/N reflects microbial processing of soil organic matter. Time since development explained soil C/N averaged over 40-cm depth (*F*_1,8.2_ = 6.8; *p* = 0.03), but yard management categories did not. However, to 10-cm depth in commercially managed yards, time since development predicted decreased soil C/N (*p *= 0.0007; r^2marg.^ = 0.80, r^2cond.^ = 0.80) but not to greater soil depths or in do-it-yourself managed yards.

Fields were a less intensively used grassland comparison with yards in our study. Since residential development 227 years prior to our sampling, fields were associated with a gain of 0.28 kg soil N m^−2^ to 40-cm depth (*F*_1,11_ = 10.44, *p* = 0.008, r^2^ = 0.49) and a decrease in soil C/N by 3.4 (*F*_1,11_ = 7.69, *p* = 0.01, r^2^ = 0.41), but were unrelated to bulk density (Fig. 5). Fields were associated with a 1.12 kg C m^−2^ gain at 20–40 cm depth over 227 years (*F*_1,11_ = 5.65, *p* = 0.04, r^2^ = 0.34), but not at other depths. In forests, that were less altered by development, soil properties were not associated with time since development.

## Discussion

### Alteration of soil carbon and nitrogen pools with residential development

There are multiple interactive controls of soil C pools across land covers. If residential development and use influence soil C pools, and are comparable across regions, this could yield similar belowground responses [51, 77]. Yet, controls of soil C pools can differ from urban core to rural edge, a dynamic socioecological and biogeophysical gradient, of variable climates, uses, and histories [8, 9, 63, 78, 79]. Legacies of these differences can create a landscape mosaic of soil C sources and sinks, that we observe in the present, and erroneously attribute to present use and cover [18, 29, 80].

We accounted for pre-development legacies to reveal the belowground consequences of development-related land conversion and present use (Fig. 1). By comparing soils that share site-specific history prior to development, we suggest that residential development and ensuing yard use can limit soil C pools for centuries. Our findings support the hypothesis that conversion-related disturbance and increasing land-use intensity drives soil C losses. Conversion of forest or field to yard disturbs soil and stimulates C release to the atmosphere [80]. This is a disturbance event from which soil C pools slowly recover [28, 81].

Soil disturbance associated with clearing, development (e.g., excavation), and use (e.g., mowing) could limit soil C storage potential. Our findings agree with the CENTURY model of yard soil C dynamics developed by Trammell and others [17] and their previous studies of the belowground legacies of development [82, 83]. Yard use that minimizes belowground disturbance could compensate for land-use conversion soil C losses, but over centuries, and this does not account for management-related C emissions [17, 52, 53]. Our research suggests that do-it-yourself residents, who mowed bimonthly to monthly and left clippings on the lawn, could compensate for soil C losses from house construction and yard establishment, but only in old yards that were not originally impacted by modern excavation and have recovered from development centuries ago (Fig. 5). Homeowners who hired commercial managers, who mowed weekly in the growing season and exported nutrients in clippings, could have suppressed recovery of yard soil C.

Following house construction, yard soil properties began to change relative to adjacent field and forest cover. Our research suggests yard soils, regardless of lawn management intensity, retained less C and N than field and forest soils since rural residential development (Figs. 2, 3, 5). Present use explained 23% of the variance in soil C pools to 40-cm depth, while site-specific pre-development legacies (LUC) accounted for an additional 27% (Additional file 1: Table S3). Present use explained only 13% of soil C pool variance to 60-cm depth, while LUC explained 14% more variance in soil C pools to 60-cm than 20-cm depth. Soil responses to residential development and present use dissipated with depth, while site-specific pre-development legacies amplified. Pre-development legacies, of anthropogenic and biogeophysical dimensions, better explained soil C pools than present use, which highlights the need to account for the history of specific places when understanding the response of slow-changing soil properties to land changes and present use [4, 84–87].

Soil N could be more responsive to present uses than soil C. Across depths, present use better explained soil N pools (r^2marg.^ = 0.39) than C pools (r^2marg.^ = 0.27) (Table 1). Site-specific pre-development legacies explained only 2% of the variation in soil N pools to 20-cm depth, but explained 16% of the variance in upper soil C pools (Additional file 1: Table S3). Present use and pre-development legacies predicted soil N concentrations (r^2marg.^ = 0.63; r^2cond.^ = 0.73) better than C concentrations (r^2marg.^ = 0.60; r^2cond.^ = 0.68) across depths. Our findings support former work in disturbed soils that showed soil N dynamics were more responsive to present use than soil C dynamics [88].

Field soil N pools were ~ 20% larger than yard and forest to 40-cm depth and associated with gains of 0.28 kg soil N m^−2^ over 227 years since development (Figs. 2, 5). Old fields, persisting for centuries, were used as pasture and fertilized by manure of grazing livestock. Nitrogen deposition could amplify at field-forest edges and further enlarge soil N pools [89]. In present use, there is limited anthropogenic N export from mown fields, relative to yards and hayed fields. Yards lose N via clipping export, denitrification, and leaching [90] which was likely uncompensated for by N inputs in our study. Nitrogen commonly limits plant productivity and C flow into soil, which could constrain yard soil C pools relative to N-rich field soils [91].

### Climatic effects on soil carbon dynamics across land-use intensity gradients

Climate alters soil C dynamics by regulating productivity and decomposition [92]. Yard microclimates could be more similar across regions and different than native uses, yielding similar C dynamics and convergence in soil C pools [51, 77, 93]. Through monitoring of one land-use cluster, we found that yard soils were an average 5.1 °C warmer and 0.11 m^3^ m^−3^ wetter than adjacent forest (Fig. 4). Warm, wet conditions could favor soil respiration, which diminishes soil C pools without C inputs and stabilization [7, 94]. Our findings from a single-site intensive monitoring study agree with former studies of microclimate along forest edges comparable to yard-field-forest gradients [95] and studies of yards across United States urban areas compared to native covers [93]. This suggests differences we observed could hold across years and land-use clusters, but warrants further study.

We present evidence of dissolved organic carbon (DOC) pulses in yard and field deep soil ~ 2 times greater than forest (Fig. 4). Dissolved organic carbon in complex with nutrients could stimulate microbes and prime soil C loss from yard and field deep soil [41, 96, 97]. However, DOC processed by microbes could lead to mineral-stabilized C [40, 98, 99], and may explain the association of field upper soil DOC, an average 30 mg L^−1^ greater than forest, with field upper soil C pools equivalent in magnitude to forest. We collected soil solution during the 2016 growing season, with 47% less precipitation than the 30-year mean [61]. Forest deep soils eventually dried until sample extraction wasn’t possible. This suggests greater evapotranspiration or leaching from forest deep soil, which could suppress microbial activity.

Macroclimate could overwhelm microclimate as a driver of soil C dynamics. In cold climates of the northern hemisphere plant productivity is temperature limited October through April. In urban and suburban areas, where plants are less temperature limited and management often compensates for moisture limitation, yard soil C pools could exceed native ecosystem soil C pools [12, 13, 45, 48, 49]. Although, these studies do not compare yards with native covers and present uses that share site-specific legacies, which are often unavailable in urbanized landscapes. In the few studies of yards in rural temperate forest, yard soil C pools have been shown to match forest soil C pools [15, 100, 101], which differs from our work. Climatic variation across regions and urban–rural gradients likely contributes to differences in yard soil C dynamics [48, 77], yet site-specific legacies are not fully unaccounted for in former research which can obscure the effect of land conversion and present use on soil C pools.

### Soil ecosystem responses to yard management

We expected soil C and N pools to respond to yard management (> 10 years) but the direction of this response after accounting for legacies was unknown. Yard management was associated with changes in soil C pools in our study, corroborating work in urban and suburban areas that show lawns accumulate soil C [12, 32, 45, 64, 90, 100]. However, after situating yards in a site-specific reference frame, our research suggests yard management has only an incremental effect on soil C pools, which remain smaller than adjacent field and forest soil C pools centuries following residential development.

Soil C pools are slow changing relative to a human life span and this trajectory could be constrained by the past [20, 102]. In models of the interaction of time since development with yard management intensity categories, site-specific pre-development legacies (LUC) did not predict yard soil N pools, but explained 27% of the variance in yard soil C pools to 60-cm depth. This suggests pre-development legacies influence yard soil C pools more than N pools. Soil N dynamics could be more responsive to yard use than C dynamics, as their cycles could be decoupled by disturbance [88]. However, soil C and N pools shared the same directional response to yard management intensity categories over time since residential development. Do-it-yourself managed yards, where clippings remained on the lawn, were associated with soil C and N pool increases. Commercially managed yards, that were mown weekly and clippings exported, were associated with soil C and N pool decreases (Fig. 5, Additional file 1: Figure S2). We do not report the magnitude of these estimates here, given the scalar mismatch between the yard management we documented (decades) and time since residential development (centuries). Nonetheless, our findings support the hypothesis that soil N inputs, via N-rich lawn grass clippings, increase soil C pools in N limited ecosystems [103, 104].

Time since development predicted decreased yard and field soil C/N (Fig. 5). Lower C/N inputs in grasslands relative to forests, and enhanced microbial processing of soil organic matter under favorable warm, wet, oxic conditions, could result in lower soil C/N. Labile, N-rich inputs of senesced grasses in yards and fields can stimulate microbes and result in stable soil C [105], particularly via belowground inputs such as deep-rooted field vegetation exudates [40]. In forests, inputs of high C/N plant biomass, and a buffered thermal environment relative to yard and field (Fig. 4), could suppress microbes and contribute to the soil C and N pool differences we observed.

In yards and fields, mowing with heavy equipment could have compacted soil. This effect was most pronounced in yards and dissipated with depth. In support of this interpretation, do-it-yourself managed yards mown monthly to bimonthly were associated with decreased bulk density over time since development to 10-cm depth, while commercially managed yards mown weekly with ride-on tractors were not. Land-use cluster did not explain variance in bulk density to 20-cm depth (Additional file 1: Table S3), suggesting similar compaction of upper soils in response to development and use regardless of site-specific pre-development legacies.

## Conclusions

We studied soil properties along yard-field-forest use-intensity gradients that shared site-specific legacies prior to development. We illustrate the need to account for anthropogenic and biogeophysical legacies, which are spatially and temporally heterogeneous, to discern the response of soils to land conversion and present use. We suggest that pre-development legacies better explain soil C pools with depth, while the effect of present use is amplified at the surface. Our work suggests building houses and establishing lawns limits soil C and N pools for centuries, which is evident when comparing yards with neighboring uses that share site-specific history prior to development. Residential development practices, such as excavation and construction, disturbs and compacts soil which could yield significant soil C loss and limit the magnitude of future soil C pools. Yard management following development disturbance can aid in soil ecosystem recovery, but our results suggest this is an incremental effect apparent only in centuries-old yards that in recent decades were mown monthly to bimonthly with clippings left on the lawn. Our research reaffirms the importance of minimizing belowground disturbance to maximize soil C storage which can influence the capacity of soils to mitigate climate change.

## Additional file

**Additional file 1.** Additional figures and tables.

## Data Availability

Data used in this manuscript is available upon reasonable request.

## Abbreviations

C: carbon
N: nitrogen
LUC: land-use cluster
SSURGO: soil survey geographic database
DOC: dissolved organic carbon
