# Correction to: Building houses and managing lawns could limit yard soil carbon for centuries

**DOI:** 10.1186/s13021-020-00145-4

**Published:** 2020-05-23

**Authors:** Morgan E. Peach, Laura A. Ogden, Eleni A. Mora, Andrew J. Friedland

**Affiliations:** 1 Ecology, Evolution, Ecosystems & Society Program, Dartmouth College, Hanover, NH 03755 USA; 2 Environmental Studies Program, Dartmouth College, Hanover, NH 03755 USA; 3 Anthropology Department, Dartmouth College, Hanover, NH 03755 USA

## Correction to: Carbon Balance Manage (2019) 14:9 10.1186/s13021-019-0124-x

Following publication of the original article [1], the authors noticed an error in the first paragraph of the Site selection subsection. The text should read, “a cool temperate forested region (mean annual 7.8 °C; 992 mm precipitation)”, instead of “a cool temperate forested region (mean annual 28 °C; 840 mm precipitation)”.
