# Supplementary material for: Building houses and managing lawns could limit yard soil carbon for centuries
Source: Carbon Balance Manag. 2019 Aug 16;14:9. doi: 10.1186/s13021-019-0124-x (PMC7227114; doi:10.1186/s13021-019-0124-x)
Supplement: Supplementary file 1 — Additional file 1. Additional figures and tables. [file 13021_2019_124_MOESM1_ESM.pdf]

Additional Figures & Tables

Additional Fig. S1 An example of 1937 aerial photography that was compared with present-day satellite imagery, then cross-referenced with interviews and archival documents to verify land-use histories. Historic aerial photographs were sourced from local libraries

Additional Fig. S2 The association of yard management with soil C pools over time since residential development to 40-cm depth. Points represent the mean of three cores per yard. Shading is the confidence interval of a significant linear mixed-effects model that accounts for pre-development legacies. Solid black lines show a significant trend when a shared y-intercept is assumed, and the dashed line a non-significant trend across all yards

Additional Table S1. Soil texture across clusters (LUC) of yards, mown fields, and forests

Additional Table S2. Differences in present land use soil properties integrated over depths after accounting for pre-development legacies

Additional Table S3. Present land use effects on soil properties integrated over depths after accounting for pre-development legacies

Additional Table S4. Time since development and yard management effects on soil properties after accounting for pre-development legacies

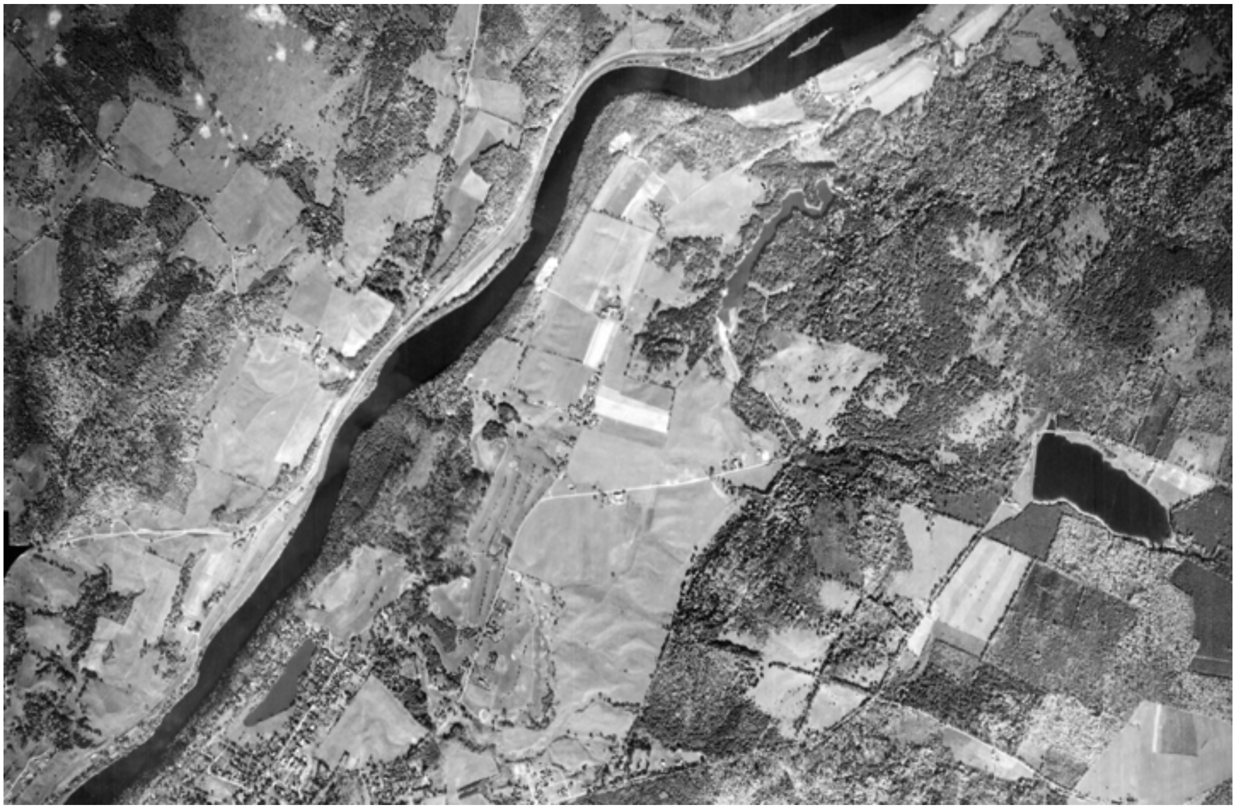

Additional Fig. S1 An example of 1937 aerial photography that was compared with present-day satellite imagery, then cross-referenced with interviews and archival documents to verify land-use histories. Historic aerial photographs were sourced from local libraries.

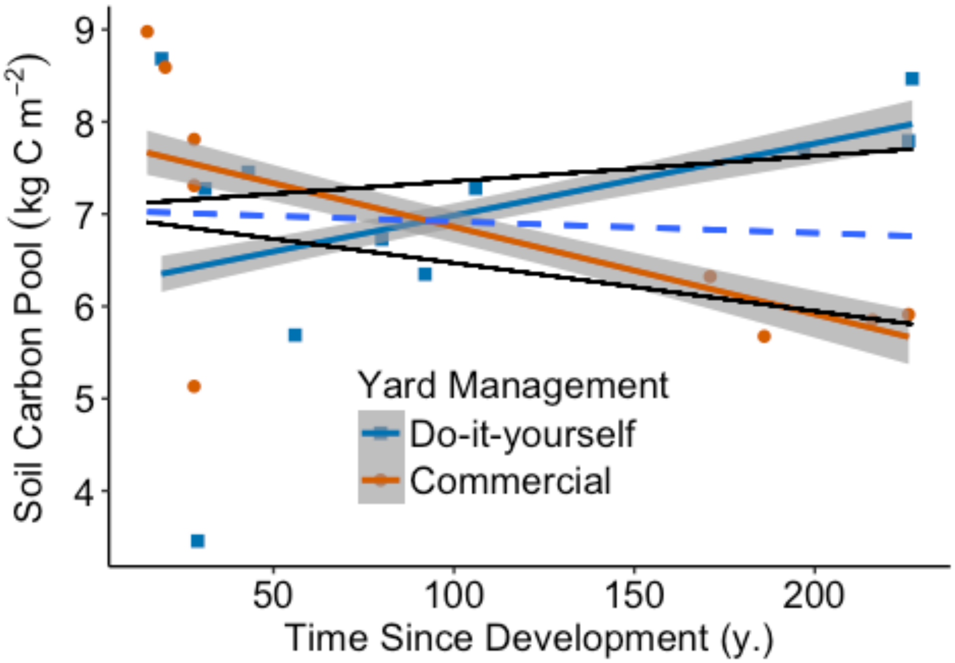

Additional Fig. S2 The association of yard management with soil C pools over time since residential development to 40-cm depth. Points represent the mean of three cores per yard. Shading is the confidence interval of a significant linear mixed-effects model that accounts for pre-development legacies. Solid black lines show a significant trend when a shared y-intercept is assumed, and the dashed line a non-significant trend across all yards.

Additional Table S1. Soil texture across clusters (LUC) of yards, mown fields, and forests

LUC	Soil series	Texture	Parent material
1	Ninigret/Dummerston	fine sandy loam	gneiss, granite, schist, or phyllite
2	Buckland/Vershire-Dummerston	loam/coarse loam	mica, schist, limestone or phyllite
3	Buckland/Glover-Vershire	loam	mica, schist, limestone or phyllite
4	Buckland	loam	mica, schist, limestone or phyllite
5	Vershire-Dummerston	coarse loam	mica, schist, limestone or phyllite
6	Buckland/Glover-Vershire-Dummerston	loam	mica, schist, limestone or phyllite
7	Buckland/Vershire-Dummerston	loam	mica, schist, limestone or phyllite
8	Groveton	fine sandy loam	granite, gneiss or schist
9	Vershire-Dummerston	coarse loam	mica, schist, limestone or phyllite
10	Agawam	fine sandy loam	gneiss, granite, schist, or phyllite
11	Hitchcock/Charlton	silt loam/fine sandy loam	granite, gneiss, or schist
12	Glover-Vershire/Hinckley	coarse loam/sandy loam	mica, schist, limestone or phyllite

Additional Table S2. Differences in present land use soil properties integrated over depths after accounting for pre-development legacies

Response	Depth (cm)	Land Use Contrast	Estimate	SE	df✦	*t* ratio	*p*-value
Soil C Pool	0-20	Yard - Field	-0.90	0.31	30.1	-2.9	0.02
		Yard - Forest	-0.91	0.32	30.7	-2.9	0.02
		Field - Forest	-0.007	0.34	29.1	-0.02	0.9
	0-40	Yard - Field	-1.60	0.41	31.3	-3.9	0.001
		Yard - Forest	-1.46	0.43	31.6	-3.4	0.005
		Field - Forest	0.14	0.46	30.6	0.3	0.9
	0-60	Yard - Field	-1.4	0.63	28.8	-2.2	0.09
		Yard - Forest	-1.8	0.65	29.2	-2.7	0.03
		Field - Forest	-0.4	0.70	26.9	-0.6	0.8
Soil N Pool	0-20	Yard - Field	-0.06	0.03	30.1	-2.16	0.09
		Yard - Forest	0.03	0.03	31.6	0.98	0.59
		Field - Forest	0.09	0.04	28.95	2.82	0.02
	0-40	Yard - Field	-0.13	0.04	31.7	-2.9	0.02
		Yard - Forest	0.003	0.04	32.6	0.06	0.99
		Field - Forest	0.13	0.05	30.7	2.7	0.03
	0-60	Yard - Field	-0.11	0.05	28.9	-2.1	0.10
		Yard - Forest	-0.01	0.05	29.3	-0.26	0.96
		Field - Forest	0.09	0.05	26.8	1.74	0.21
Soil C/N	0-20	Yard - Field	0.28	0.65	42	0.4	0.9
		Yard - Forest	-2.98	0.67	42	-4.4	0.0002
		Field - Forest	-3.3	0.74	42	-4.4	0.0002
	0-40	Yard - Field	2.8	1.65	42	1.7	0.2
		Yard - Forest	0.07	1.7	42	0.04	0.9
		Field - Forest	-2.7	1.9	42	-1.4	0.3
	0-60	Yard - Field	1.9	1.3	28.7	1.4	0.3
		Yard - Forest	0.01	1.37	29.7	0.008	1
		Field - Forest	-1.9	1.4	26	-1.4	0.4
Bulk Density	0-20	Yard - Field	0.19	0.05	42	3.7	0.002
		Yard - Forest	0.38	0.05	42	7.2	<0.0001
		Field - Forest	0.19	0.06	42	3.4	0.005
	0-40	Yard - Field	0.14	0.05	29.0	2.6	0.04
		Yard - Forest	0.32	0.06	30.5	5.8	<0.0001
		Field - Forest	0.18	0.06	27.8	3.0	0.01
	0-60	Yard - Field	0.03	0.05	27.8	0.7	0.79
		Yard - Forest	0.20	0.05	28.4	3.7	0.002
		Field - Forest	0.17	0.05	25.6	3.1	0.01

✦degrees of freedom by Satterthwaite approximations. Contrasts of the model: response ~ land use + (1 | land-use cluster) with Tukey adjustment of *p*-values. Bulk density and soil C/N are averaged over depth increments.

Additional Table S3. Present land use effects on soil properties integrated over depths after accounting for pre-development legacies

Response	Depth (cm)	*F* value (df✦)	*p*-value	marginal r^2✦	conditional r^2✦
Soil C Pool	0-20	6.1 (2, 30)	0.006	0.18	0.34
	0-40	9.6 (2 ,31.2)	0.0005	0.23	0.5
	0-60	4.3 (2, 28.2)	0.02	0.13	0.43
Soil N Pool	0-20	4.3 (2, 30.3)	0.02	0.16	0.18
	0-40	5.1 (2, 31.7)	0.01	0.17	0.25
	0-60	2.5 (2, 28.3)	0.097	0.08	0.34
Soil C/N	0-20	12.5 (2, 42)	<0.0001	0.36	0.36
	0-40	1.6 (2, 42)	0.2	0.07	0.07
	0-60	1.3 (2, 28.1)	0.3	0.05	0.17
Bulk Density	0-20	26.6 (2, 42)	<0.0001	0.55	0.55
	0-40	17.0 (2, 29.2)	<0.0001	0.43	0.45
	0-60	7.7 (2, 27.2)	0.002	0.23	0.44

✦denominator degrees of freedom by Satterthwaite approximations. Contrasts of the model: response ~ land use + (1 | land-use cluster). Marginal r^2 represent the fixed effect of land use and conditional r^2 include the random effect of land-use cluster. Bulk density and soil C/N are averaged over depths.

Additional Table S4. Time since development and yard management effects on soil properties after accounting for pre-development legacies

Response	Factor	*F* value (df✦)	*p* value	marginal r^2✦	conditional r^2✦
Soil C Pool	Management	1.9 (1, 12.6)	0.2	0.24	0.30
	House Age	0.04 (1, 58.3)	0.8		
	Depth	16.5 (1, 65.3)	0.0001		
	Management:House Age	5.4 (1, 12.3)	0.04		
Soil N Pool	Management	1.5 (1, 12.5)	0.24	0.38	0.43
	House Age	1.2 (1, 58.9)	0.28		
	Depth	39.9 (1, 64.9)	<0.0001		
	Management:House Age	5.0 (1, 12.0)	0.04		
Soil C/N	Management	0.01 (1, 12.0)	0.9	0.18	0.22
	House Age	8.8 (1, 67.4)	0.004		
	Depth	12.1 (1, 61.5)	0.0009		
	Management:House Age	0.5 (1, 11.2)	0.49		
Bulk Density	Management	0.36 (1, 9.1)	0.56	0.13	0.20
	House Age	3.8 (1, 54.0)	0.06		
	Depth	0.08 (1, 62.9)	0.77		
	Management:House Age	3.7 (1 , 8.9)	0.09		

✦degrees of freedom by Satterthwaite approximations. Marginal r^2 represent the fixed effect of land use and conditional r^2 include the random effect of land-use cluster. Yards were either mown weekly and clippings exported (n = 9) or mown bimonthly to monthly and clippings remained (n = 11). Results from model: response ~ house age*management + depth + (1 | land-use cluster). House age was a proxy for time since development.
